# Supplementary figures and images for: Structural characteristics of lipocalin allergens: Crystal structure of the immunogenic dog allergen Can f 6
Source: PLoS One. 2019 Sep 16;14(9):e0213052. doi: 10.1371/journal.pone.0213052 (PMC6746357; doi:10.1371/journal.pone.0213052)

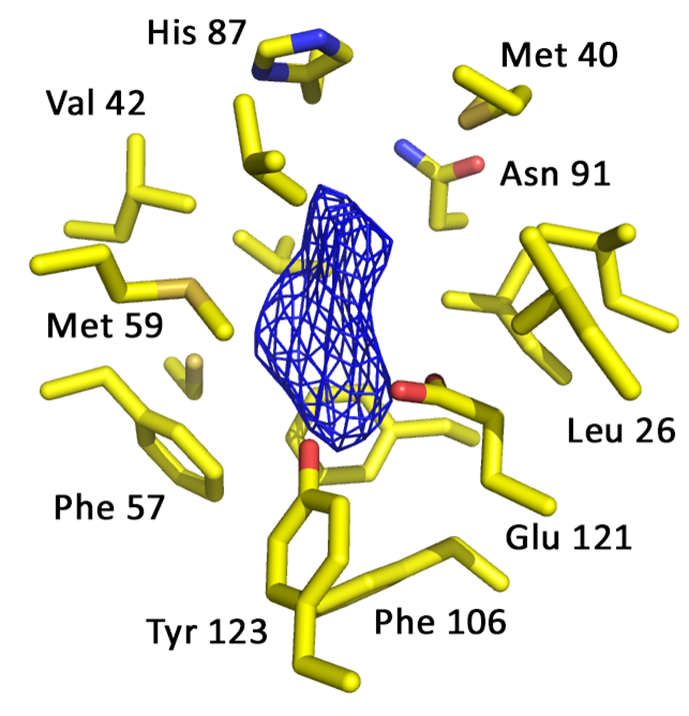

Supplement: S1 Fig — The binding surface is predominantly lined by hydrophobic amino acids. (TIF) [file pone.0213052.s001.tif]
