# Supplementary material for: Structural characteristics of lipocalin allergens: Crystal structure of the immunogenic dog allergen Can f 6
Source: PLoS One. 2019 Sep 16;14(9):e0213052. doi: 10.1371/journal.pone.0213052 (PMC6746357; doi:10.1371/journal.pone.0213052)
Supplement: S1 Table — (DOCX) [file pone.0213052.s002.docx]

| **Surface residues** | 148 (92%) |  |  |  |
| --- | --- | --- | --- | --- |
| **Symmetry Operation** | y, x,-z | -y+1, x-y+1,  z+1/3 | -x, -x+y,  -z+1/3 | x-y, -y+1,  -z-1/3 |
| **Interface residues** | 27 (17%) | 17 (10%) | 16 | 4 (3%) |
| **Buried ASA**  **(Å^2^)** | 736 (9.4%) | 510 | 420 (5%) | 69 (1%) |
| **Hydrogen bonds** | 14 | 2 | 0 | 1 |
| **Salt bridges** | 0 | 0 | 0 | 0 |
| **Complex Formation Significance Score (CSS)** | 0.1 | 0 | 0 | 0 |
| **Total ASA** | 7826 |  |  |  |

**S1 Table.** **The Can f 6 monomer-monomer surface interfaces within the crystal packing generated in PISA.**
